# Autonomic care platform for optimizing query performance

**DOI:** 10.1186/1472-6947-13-120

**Published:** 2013-10-27

**Authors:** Kristof Steurbaut, Steven Latré, Johan Decruyenaere, Filip De Turck

**Affiliations:** 1 Department of Information Technology, Ghent University - iMinds Gaston Crommenlaan 8 Bus 201, 9050 Gent, Belgium; 2 Department of Mathematics and Computer Science, University of Antwerp - iMinds, Middelheimlaan 1, 2020 Antwerp, Belgium; 3 Department of Intensive Care, Ghent University Hospital, De Pintelaan 185, 9000 Gent, Belgium

## Abstract

**Background:**

As the amount of information in electronic health care systems increases, data operations get more complicated and time-consuming. Intensive Care platforms require a timely processing of data retrievals to guarantee the continuous display of recent data of patients. Physicians and nurses rely on this data for their decision making. Manual optimization of query executions has become difficult to handle due to the increased amount of queries across multiple sources. Hence, a more automated management is necessary to increase the performance of database queries. The autonomic computing paradigm promises an approach in which the system adapts itself and acts as self-managing entity, thereby limiting human interventions and taking actions. Despite the usage of autonomic control loops in network and software systems, this approach has not been applied so far for health information systems.

**Methods:**

We extend the COSARA architecture, an infection surveillance and antibiotic management service platform for the Intensive Care Unit (ICU), with self-managed components to increase the performance of data retrievals. We used real-life ICU COSARA queries to analyse slow performance and measure the impact of optimizations. Each day more than 2 million COSARA queries are executed. Three control loops, which monitor the executions and take action, have been proposed: reactive, deliberative and reflective control loops. We focus on improvements of the execution time of microbiology queries directly related to the visual displays of patients’ data on the bedside screens.

**Results:**

The results show that autonomic control loops are beneficial for the optimizations in the data executions in the ICU. The application of reactive control loop results in a reduction of 8.61% of the average execution time of microbiology results. The combined application of the reactive and deliberative control loop results in an average query time reduction of 10.92% and the combination of reactive, deliberative and reflective control loops provides a reduction of 13.04%.

**Conclusions:**

We found that by controlled reduction of queries’ executions the performance for the end-user can be improved. The implementation of autonomic control loops in an existing health platform, COSARA, has a positive effect on the timely data visualization for the physician and nurse.

## Background

With an increased growth of clinical support services and data sources, clinical information service platforms are becoming more and more complex. The emergence of medical devices, which monitor and collect data at high frequency, the availability of data in numerous databases and the increased utilization of the electronic patient data to support physicians’ clinical decisions, demand a high speed of data processing. Physicians and nurses put trust in electronic medical records to evaluate the patients’ conditions and to treat patients by taking therapeutic decisions. Slow data retrievals force the physician to wait longer for results of the current state of the patient. Due to the large amount of data variables and hence a high number of database queries, manual maintenance operations are no longer possible. For example, manually disabling time-consuming non-priority data retrievals in case of high load on the system is difficult. Moreover, in the medical environment the contents of the database is constantly changing with inserts of medical data or updates of existing values from medical devices which monitor the patient at high frequency or analyse the patients’ laboratory samples. Despite system administrators’ efforts to maintain critical health systems, symptoms of data slowdown cannot be detected in time and actions cannot be taken quickly enough to prevent performance decrease or system failure. This leads to a degradation of service quality and availability. Therefore, the manual reaction to such slow processes undermines the robustness and performance of the complete system.

### Adding autonomic capabilities to the COSARA system

The autonomic computing paradigm aims to develop systems capable of self-management, which make decisions on their own and respond with appropriate actions on system failures or optimizations. This concept is in analogy with the autonomic nervous system, which manages our vital functions in the body without conscious directions [1]. In autonomic computing, an autonomic manager implements control loops in which the managed element and the environment is monitored, data is analyzed, and actions are taken if components are in an undesirable state. It envisions a self-aware software system. In this article, we extend the existing COSARA health care platform with autonomic components. COSARA is an infection surveillance and antibiotic management service platform for the Intensive Care Unit (ICU) [2]. We propose extensions to COSARA by introducing multiple autonomic control loops. The reactive control loop takes an immediate action when slow data query executions are detected. In the deliberative control loop the decision to act is evaluated in an anomaly detection algorithm with detection of anomalies in the execution times of data retrievals. Anomalies are also predicted in the reflective control loop by detecting temporal periods with slow performance. A detailed analysis has been performed based on real-life data logs from the COSARA platform in the ICU of Ghent University Hospital. This article is structured as follows. In Section 'Related work’, an overview of autonomic computing architectures is presented and specific models from the health care domain are explored. The problem of managing COSARA data queries is thoroughly explained in Section 'Problem statement’. The extended architecture of the COSARA service platform is presented in Section 'Architecture’. Section 'Design of FOCALE-based control loops in the COSARA architecture’ describes the multiple control loops, which enhance performance of data queries. This includes a reactive loop, a deliberative loop that takes a decision by executing an anomaly detection algorithm and a reflective control loop that takes a proactive approach by detecting temporal patterns. Subsequently, the optimizations are evaluated in detail in Section 'Results and discussion’. Finally, Section 'Conclusions’ presents the conclusions of this paper.

### Related work

Although autonomic management has received attention in enterprise wide network platforms, only a limited number of studies apply autonomic management to health care platforms. In this section we examine related work in both domains.

#### Autonomic management in health care

Autonomic computing has already been applied in body area networks in health care. On-body sensors monitor the patient’s vital functions such as heartbeat, body temperature or electrocardiogram (ECG) in a body area network and transmit the signals to a processing unit. Since this equipment is hard to maintain by its developers, the system should adapt automatically to changes. The telemonitoring applications that use continuous monitoring of patients’ health conditions require the self-management ability that autonomic systems propose [3]. In [4], an event service for autonomic management support for e-health systems is proposed using Self-managed cells (SMCs). SMCs are autonomic systems that are able to add or remove components, detect failures of sensors automatically and adapt the system. In [5], it is described as an architectural pattern to provide Autonomic Management of Ubiquitous e-health Systems (AMUSE). The system needs to be self-configuring and self-managing with limited user interaction and autonomously adapts to changes in user activity, device failure and service addition. The SMC consists of an event bus, for communication between devices and management services, a discovery service and policy service [4]. The policy service specifies the adaptation strategy (adaptation, authorization policies and event-condition-action rules) whereas the discovery service implements the protocol to search and integrate new devices in the SMC and maintains the connections. Changes in the environment are indicated by events, which trigger policies in the policy service and hence perform the action [6]. In the used publish-subscribe mechanism, messages are published on the event bus and delivered to its subscribers, instead of directly delivering the message. In the VESTA system, the AMUSE system is extended with security support and policy management for authentication and access control [6]. In [7], an autonomic model for the management of health care applications has been presented, adopting the MAPE control loop. This control loop consists of monitor, analyse, plan and execute phases and interacts with a knowledge layer. The model has been used to assure process quality of the medical information system and as supervisor of the compliance of medical decisions with the protocols [7]. It has been applied for the treatment planning of diabetes. The prediction service in this system, which predicts the patient’s diagnosis using multiple regression, is implemented as a web service. Autonomic computing has also been applied in the hospital’s emergency department to maintain optimal quality of service and optimize performance of operations [8]. These departments suffer from a high workload due to an increased demand on health resources and a limited clinicians staff. Sensors monitor the state of the environment (for example by using optical sensors, radio-frequency identifiers (RFIDs) and counters for people and workload). However, related work in papers covering autonomic health care mainly concentrates on the architectural models. To the authors best knowledge, no previous studies have been conducted which design, implement and evaluate autonomic control loops in the intensive care, with the aim to increase performance of data retrievals.

#### Autonomic architectures

Autonomic architectures have been applied in industry systems to find early indications of failures and to investigate fault causes. The MAGNETO project [9](2010) focuses on probabilistic fault diagnosis to find the cause of service problems, such as service degradation and service breakdowns, in home area networks. The causes of network failures and observed network variables are modeled in a bayesian network which can infer the probability of the cause of a service failure. Several initiatives for building autonomic network architectures have been investigated in [10], consisting of hierarchical architectures, flat autonomic architectures and self-organizing networks. One of the hierarchical architectures is the Autonomic Internet project (AutoI) which deals with the autonomic management for the future internet in which autonomic management is applied to the management of virtual resources. The Component-ware for Autonomic, Situation-aware Communications, and Dynamically Adaptable Services (CASCADAS) deals with the development of an autonomic framework for creating, executing, and provisioning situation-aware and dynamically adaptable communication services [11]. In [12], an anomaly detection framework was proposed to provide techniques to analyze and detect anomalies in runtime data of cloud systems by applying (i) data transformation, (ii) feature selection, (iii) outlier detection. Anomalies or outliers are patterns in data that do not conform to a well defined notion of normal behavior [13]. Detection techniques have been developed to find these patterns which often represent exceptions, indications of system failure or interesting data which should lead to actions. Anomaly detection has been used in a variety of domains such as fraud detection of credit cards, fault detection in safety critical systems, insurance or health care, military surveillance using a diversity of techniques such as statistical methods, data mining, machine learning [13]. Rabatel et al. [14] addressed the problem of maintaining complex systems through preventive maintenance which detects abnormal behavior though collecting sensor data and analysis and found that these anomalies may lead to failure. In our case, we want to detect low data query performance.

#### Control loops

**Figure 1:**
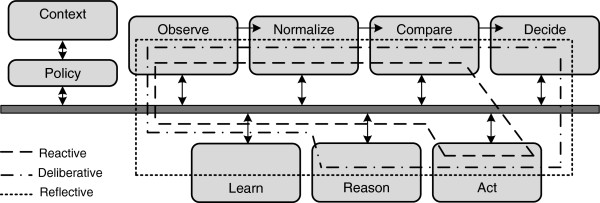
**The FOCALE control loops.** FOCALE features several dynamic control loops: reactive, deliberative and reflective control loops. The control loop is formed by the following steps: Observe, Normalize, Compare, Decide, Act, Reason and Learn. The different types of control loops are shown on the figure. The reactive control loop takes immediate action based on external observations. The deliberative control loop uses long and short memory to create an action plan. The reflective control loop uses the learning component to take preventive actions in the future.

The core of an autonomic system is the Autonomic Manager (AM) which includes one or more control loops that monitor the resources, analyze the data to determine if the status is normal or if adaptations are needed. If actions are needed, these are planned and executed. This type of control loop maps the sequence: Monitor, Analyze, Plan and Execute (MAPE), as introduced by IBM [1]. Since the original proposal of autonomic computing by IBM several new control loops have been proposed extending the MAPE control loop. One of the most widely used set of control loops is those of the FOCALE architecture. The FOCALE autonomic architecture (Foundation - Observe - Compare - Act - Learn - rEason) consists of advanced control loops with extended capabilities for knowledge use and learning [15]. The FOCALE control loops have served as basis for the CASCADAS architecture and have been successfully applied to, amongst others, fault management [16] and management of the home network [17]. Because of its popularity, we use the FOCALE control loops as the basis of our autonomic manager and discuss its details in the remainder of this section. The FOCALE control loops are shown in Figure 1. The components in FOCALE are connected by an enterprise service bus (ESB), an event-driven message broker that supports different types of knowledge and performs processing before delivery. FOCALE uses a combination of information/data models and ontologies [15]. The FOCALE control loops are formed by running through a number of steps. In the Observe step, monitored observations are retrieved and fed to a model-based translation process of the Normalize step. The process facilitates the translation of device specific information into a normalized form. This normalized data is then analyzed to determine the current state of the system. Subsequently the current state is compared to the desired state of the system in the Compare step. In the Reason step, a reasoning algorithm evaluates the decisions and in the Learn step future predictions are made. FOCALE features several dynamic control loops, which can be classified into three categories that also resemble actions identified in mental concepts of the human brain [18]. More specifically, FOCALE allows to define three different types of control loops that each have an increasing level of cognitive capabilities. Reactive control loops take immediate responses based on external stimuli. They react in order to carry out one or multiple goals. Additionally, shortcuts can be taken in order to perform high-priority and urgent tasks. The reactive control loops run at the highest frequency and circumvent the decide, reason and learn components. Deliberative control loops receive data from and can send commands to the reactive processes. They use long and short term memory to create more elaborate plans of action. The deliberative loops run at a lower frequency and circumvent the learning component. Finally, the reflective control loops supervise the deliberative processes. They study decisions made in the past, and analyse them. The conclusions are then used to prevent sub-optimal actions from being taken again in the future. The reflective loops run at the lowest frequency.

The major advantage of the above described FOCALE cognitive control loop approach is its high variety in offered pro-activeness. As it consists of multiple control loops with different characteristics (reactive, deliberative and reflective) urgent tasks can be a less complex control loop (e.g., a reactive variant) and iteratively improved later on by a more complex control loop (i.e., a deliberative or reflective control loop). The FOCALE cognitive control loops have mainly seen an implementation in the area of network and service management. In previous work, we applied it to manage multimedia services by extending it with semantic capabilities [19]. In this work, the focus was on combining different elements to jointly manage a service such as the streaming of multimedia in a computer network. Choi et al. have embedded the FOCALE control loop in their HiMang architecture [20]. Their focus is more on the architectural aspect of the FOCALE architecture and less on the algorithmic implementations of the different control loops: they investigate the integration with policies and information models. Moreover their application domain is different to our approach as they study cloud-based networks, Quality of Service management and fault management. Kim et al. have implemented the FOCALE control loops to manage OpenFlow-based networks (i.e., the protocol that steers the Software-Defined Networking paradigm) [21]. Their solution uses the FOCALE control loops to allow setting up and maintaining paths in OpenFlow, even if unexpected link failures occur. Their approach focuses on maintaining datapath connectivity, while our approach has query optimization and management as primary goal. For this reason, the algorithmic approach is completely different. The same authors have also implemented the FOCALE control loops to prioritize and group alarms raised by a network management system [16]. As this corresponds with a classification problem, it is more related to our approach. However, we use a semi-supervised learning approach through an anomaly detection algorithm, while they propose a more static rule-based approach.

#### Control theory approaches to query optimization

In this article, we propose an autonomic management approach to query optimization in a health information system. We present anomaly detection based algorithms that implement the aforementioned FOCALE cognitive control loops. The concept of control loops stem from control theory [22], which is a paradigm that allows to dynamically manage a system based the maximization of an objective and periodic or continuous feedback from the managed system. In the past, control theory has been successfully applied to many application domains (e.g., resource allocation [23], web server management [24], application server management [25]). Typically these control loop approaches try to predefined service level objectives. Parekh et al. describe a methodology for designing control loops for managing service level objectives in performance management [26]. Through the design of a statistical model, fit to historical measurements, they avoid requiring large and complex mathematical models. Our proposed system, and more specifically the reflective and deliberative control loops, uses the same approach: by detecting temporal patterns in historical data optimal actions are learned without requiring a model of the complete system. Hellerstein et al. also introduced the concept of such a statistical approach to predict future demands of software systems (e.g., a Web Server) [27]. Our approach extends this idea and introduces different levels of learnings.

Optimization of query processing often re-uses concepts of control theory and has mainly been studied in the context of grids [28]. More recently, with the growing attention towards Big Data, new application domains of this research have been found [29]. These approaches typically focus on large scale and distributed databases. As such, they are complementary to our approach as they can be used if the scale of the database system itself increases (e.g., introducing a higher level of replication). Also in the area of query optimization, dynamic management approaches have been proposed. Paton et al. [30] propose an adaptive query processing algorithm with the same goal as our approach: reducing the overall response times of query processing. They focus mainly on the joint optimization of multiple queries as they are often grouped, requested by a single user. Park et al. present an approach where queries consisting of multiple joins are optimized [31]. Their main approach consists of developing multiple candidate processing plans and only selecting the best plan after some initial pre-processing steps. Avnur et al. focus on adaptive query processing in large-scale and federated databases [32] by continuously reordering joins inside a single query based on the observed - and highly dynamic - response times of subqueries in such a federated database. The above approaches can be seen as complementary solutions to our approach as they focus on the optimization of specific queries. As such, they investigate the structure of each query but are agnostic to the application demands regarding these queries. Instead, we focus on the application demands and allow the application to prioritize the queries based on the application logic and user expectations. By disabling less important queries we can already considerably improve the response time. This response time could even be improved further by applying the aforementioned techniques.

More generally speaking, the problem investigated in this article relates to dynamic scheduling in a resource constrained environment. In this area, several well-known techniques exist for scheduling requests (in our case: queries) such as earliest deadline first, first come first served, etc. We refer to the work of Suresh et al. for a complete survey of these approaches [33]. Also in grids [34] and, more recently, cloud systems [35] job and application scheduling algorithms have been successfully applied. These techniques typically have a very broad application domain but also often parameters to be set (e.g., the deadline of every request). In our approach, these techniques can be used as an alternative to the current applied optimization action: the disablement of less important queries. We chose not to use these actions as we observed little effect in performance compared to a higher complexity in the configuration of the algorithm.

In summary, compared to the current state of the art, our approach is novel for the following reasons. First, the adopted three-layer approach, inspired by the FOCALE cognitive control loops, provides an important flexibility in the level of proactiveness that can be achieved. Compared to other data management approaches, we are able to quickly react to local problems and at the same time carry out more complex optimizations on a larger time scale. Second, to the best of the authors’s knowledge, our approach is the first that implements these control loops for data management. As such, the proposed anomaly detection algorithms and their integration with the three different loops, are completely novel and fundamentally different from previous approaches, which focused more on network and service management.

## Methods

### Problem statement

The COSARA platform is a platform for infection surveillance and antibiotic management in the intensive care [2]. It is being used by physicians and nurses at the ICU of Ghent University Hospital, as part of the clinical workflow. COSARA is designed as a service oriented architecture and manages the antibiotic consumption and infection related information in the ICU [2]. The COSARA system collects data from the laboratory, the clinical information system, and its own historical COSARA-database, processes these data, and presents the information or medical advice on a bedside computer, desktop at the physician’s office or at a mobile device.

The most frequently consulted data on the bedside computers consists of the patient’s clinical values and the microbiology results in this ICU. COSARA has a module offering a clinical overview with the values of temperature, white bloodcell count (WBC), thrombocytes, organ failure score, and prescribed antibiotics, and a module giving all microbiology results (samples with cultures, antibiogram and blood analyses). The COSARA system is designed in such a way that each time a physician or nurse requests the clinical values of a patient, all necessary information is requested through a series of queries (called a query group). This typically results in a burst of queries, each time a patient record is requested.

**Figure 2:**
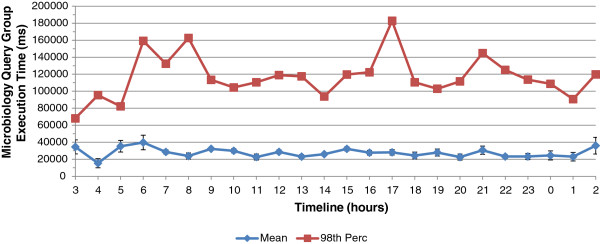
**Delay of COSARA microbiology query group over 24h window: average execution time and 98th percentile.** This figure shows the delay of the COSARA microbiology query group, consisting of microbiology samples, cultures, antibiograms and analyses, over a 24h time window: the average execution time and 98th percentile. It corresponds to the page load of the microbiology module in the COSARA application. As shown, the highest peaks of the 98th percentile show execution times of 182 s, 162 s, 59 s in a 60 minutes time frame, whereas average execution times are observed around 27 s execution time.

Besides these queries who feed the displayed modules, other COSARA queries update data in the background. On an average day, approximately 2 million COSARA queries are executed, with an average of approximately 85,000 queries per hour. The growing popularity of the COSARA application affects the data response times in the client. With more queries being executed simultaneously, the execution time of data retrievals increases and delays are noticed. This is illustrated in Figure 2, which shows the average execution time and 98th percentile required to retrieve the microbiology samples, cultures, antibiogram and analyses on the microbiology module. It shows the page load of the microbiology module in the COSARA application. As shown, the highest peaks of the 98th percentile show execution times of 182 s, 162 s and 59 s in a 60 minutes time frame, whereas average execution times are observed around 27 s execution time. As physicians depend on the application to support their clinical decision, high delays have to be prevented. The human operator is unable to guard the execution of the 150 different query types in the COSARA database. Therefore, potential delays in the data retrieval should be prevented autonomously by system components.

### Architecture

**Figure 3:**
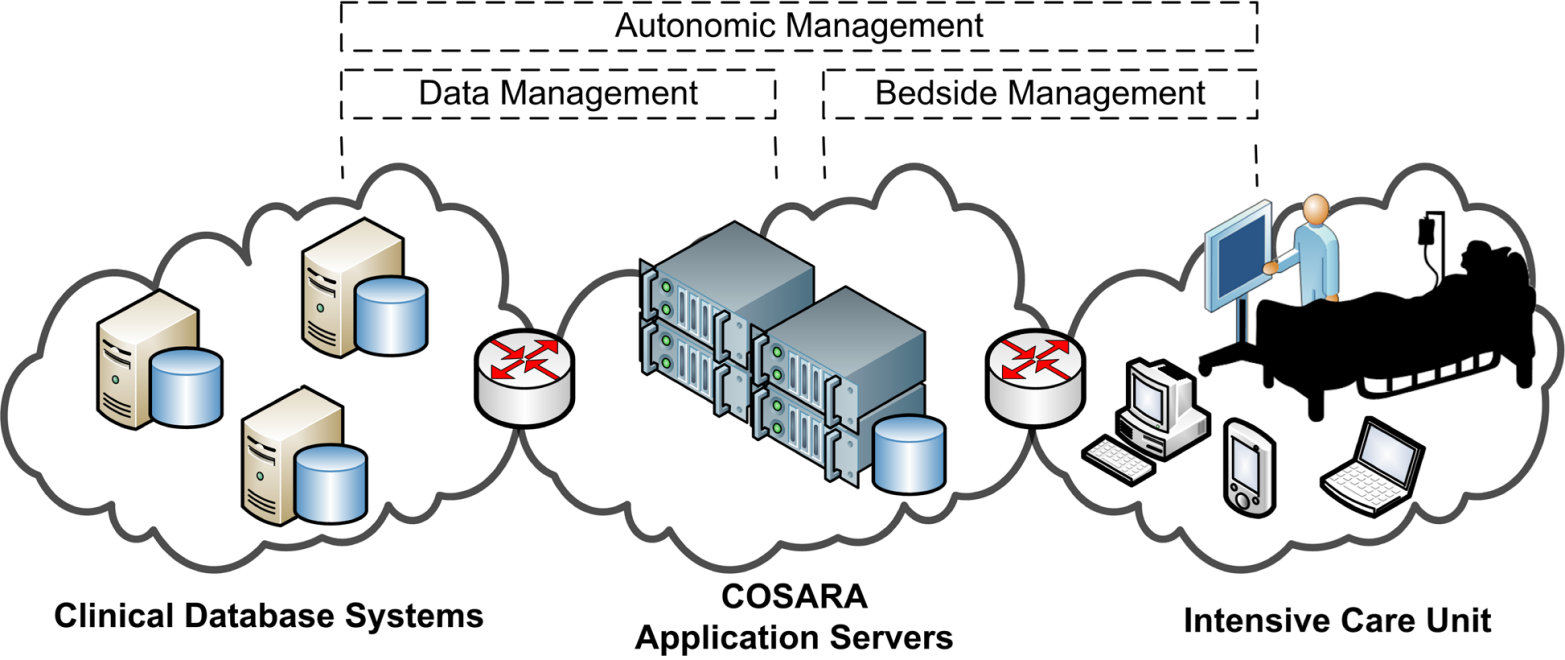
**Overview of the COSARA setting in ICU.** This figure illustrates the domain where autonomic management is applied in health care. It includes the data management of timely bedside procedures and the management of data retrieval and processing. On the bedside screen the retrieval time for the microbiology data should be optimized. On the server side, the data management with underlying query executions on the database should be optimized further. The bedside and serverside management are linked with each other.

**Figure 4:**
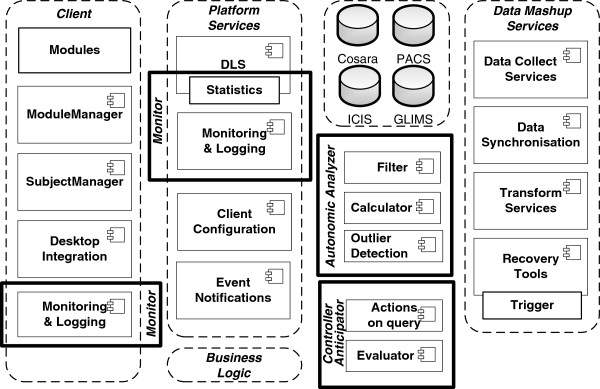
**The extended COSARA architecture.** The COSARA architecture has been extended with components to enable autonomic management. The architecture was a service oriented architecture consisting of layers for presentation, business processing and data persistence. As shown on the figure, the added components include Monitor components, Autonomic Analyzer, and Controller and Anticipator. These components correspond to the steps in the FOCALE architecture.

The system should identify, manage and thus prevent the performance issues autonomously by reacting quickly on behavior changes in the system components. These changes can result, for example, from high utilization or an increased frequency of data retrievals. To make appropriate and reliable decisions, the concern is to possess data that is accurate enough, timely enough and consistent enough [36]. Figure 3 illustrates the domain where autonomic management is applied in health care: (i) the data management of timely bedside procedures and (ii) the management of data retrieval and processing. The COSARA service-oriented architecture consists of layers for presentation, business processing and data persistence [2]. We extended the architecture with components, as shown in Figure 4. The client is designed in a modular way (Modules and Module Manager) using the OSGi technology as basis. Modules can be added, removed or updated on all bedside clients by changing the configuration on the server. Both in client and platform services, *monitoring and logging* components are added in order to be able to track the state of bedside client and server-side components. The Data Lookup Service (DLS), which forms the interface towards the data sources, is extended with a statistics component. The DLS logs every data access and includes the invocation time, the logical query name and the query’s execution time (in milliseconds). The DLS executes all queries (antibiotics, laboratory, microbiology, infection-related queries) on the different data sources (the laboratory database GLIMS, the intensive care information system (ICIS) or the COSARA database). The *autonomic analyzer* ensures that the monitored data and logs are examined dynamically (as detailed in the control loops). In the iterative design and evaluation of COSARA, we already started with the addition of recovery tools and limited detection mechanisms, but the loop was not closed and the human administrator had to take action. The analyzer now detects performance decreases in the execution of queries and instructs the *Controller*-*Anticipator* to respond and adapt the query executions autonomously to optimize the quality of service. In the implemented control loops, the Controller-Anticipator responds by temporarily disabling less important queries, which are typically part of the query group stemming from a physician’s or nurse request for a patient record or background queries.

**Figure 5:**
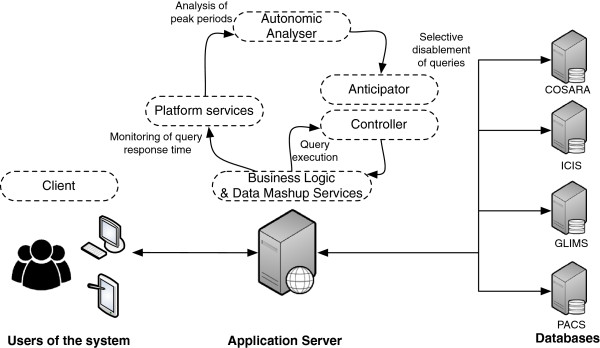
**Mapping of the functional architecture to the physical topology of the application.** This figure depicts how the functional architecture of Figure 4 maps to physical locations. Moreover, it shows how different functional modules in the architecture are connected with each other to form an autonomic control loop. Finally, it clarifies how response times are measured on the application server by interacting with the databases.

Figure 5 illustrates how this functional architecture is mapped to the physical infrastructure. As shown, the business logic, deployed at the application server responds to user queries and/or background tasks (e.g., periodic maintenance). This results in the execution of a series of queries, which are forwarded to the different databases, via the controller. Each query execution has a certain response time. These response times are monitored and aggregated: for each query execution the delay before query execution and return of the result is monitored and grouped in the according query group. A query group is a group of queries that are all executed as a reaction to a user operation (e.g., the retrieval of a patient’s record). Note that the monitoring of these response times occurs completely locally. As discussed above, the result of these measurements are analysed, which can potentially lead to the selective disablement of queries.

Note that we chose to disable the queries instead of opting for more complex scheduling algorithms as discussed in Section 'Related work’. The reason for this is the following: priority-based algorithms would assign lower priorities to less relevant queries. The particular problem tackled in this manuscript, however, has to deal with long peak periods. As during peak periods, new high priority queries are constantly being introduced, low-priority queries are continously ignored, until their relevance becomes obsolete. As a result, their execution becomes useless and the effect is similar to action we chose, which is disabling the less important queries from the start.

### Query selection process

As described above, the approach presented in this article aims at reducing the response times of queries during peak hours in the COSARA architecture by temporarily disabling less important queries. A first step in achieving this is the selection of queries, which have lower priority for the users of the COSARA system. This requires domain knowledge about the application. In this section, we first describe how this knowledge is modelled and subsequently present an algorithm for selecting candidate queries.

#### Knowledge model

We model the domain knowledge of the COSARA application using RDF^a^, which is a standardized model for data interchange. One advantage of RDF is that models for well-known and generic concepts are available in RDF or the ontology language OWL^b^, which uses RDF-based serialization. For this reason, we re-use existing models for incorporating the relevant knowledge as much as possible.

More specifically, we use the IntelLEO Workflow ontology^c^ to model how users and background processes interact with the COSARA system. The IntelLEO Workflow ontology defines concepts such as roles and users, and allows defining a sequence of activities and tasks. As will be described below, this is used to define the clinical decision process of the users in combination with the COSARA system. In defining these workflows, we often need to refer to medical terminology. Therefore, we use the Galen ontology [37], which defines this terminology and their links. Finally, we need to model the application specific knowledge linked with the COSARA application (e.g., which queries are executed when). We do this by linking newly defined concepts to the tasks and activities defined in the workflow. More specifically, we allow a task to refer to a set of queries: each query can have subqueries and has attributes such as a value defining its complexity and priority.

**Figure 6:**
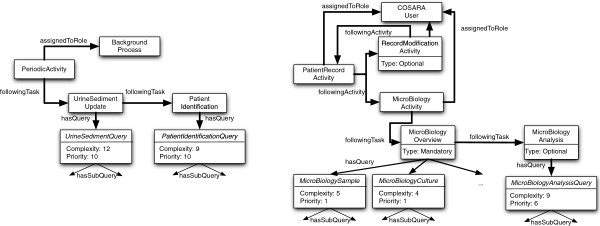
**Knowledge modelling of two illustrative workflows in the COSARA system: one periodic background process and one user-triggered process.** This figure shows an example of how domain knowledge about the COSARA application, and more specifically the interaction between workflow and query optimization, is modeled in our approach. Two illustrative workflows are shown: one corresponding with an automated background process and one corresponding with a sequence of activities carried out by a COSARA user.

Figure 6 provides an example of how the knowledge is modelled for the COSARA application. For the sake of readability, we only show the link with the workflows and queries, not with the Galen ontology. Here we defined two workflows: one periodic activity belonging to a background process in the application and one patient specific activity, which is carried out by a COSARA user (either a physician or nurse). The periodic activity consists of several maintenance tasks, which are sequentially executed. Each task has its own query, with multiple subqueries (not shown in the figure). As these are maintenance task, their priority is small (note that a high priority value, corresponds to a low priority). The user-triggered activity details how a user accesses microbiology results in the COSARA application. The user is first shown a summarized view on the patient’s details. To create this view several queries are required. The user then has the option to either modify the patient’s details (after which he returns to the patient overview) or to get an overview of the microbiology details. This microbiology activity consists of one mandatory task (with several queries required to create this view) and one optional task, which performs the analysis of the results.

#### Identification of candidate queries for disablement

In this section, we present an assisted algorithm for identifying less important queries. The algorithm ranks the different queries in the COSARA system based on their importance for the users. This ranking is then given to an administrator as an aid in selecting the queries that can be disabled when a scarcity in resources occur.

Overall, the ranking is done based on three factors (i) the visibility of the queries in the application, (ii) the query’s complexity and (iii) the importance of the queries in the clinical decision process. The algorithm provides a formal implementation of a manual heuristic, which was initially carried out by the administrator of the COSARA system. An overview of the algorithm is given in Algorithm 1. As shown, the algorithm iterates on all different activities and corresponding tasks and queries in the workflow. Each query is given an initial rank of 1, which corresponds to the highest importance. However, several factors can increase the rank value (i.e., lower the query’s importance). For example, background processes and optional activities or tasks are penalized with a factor (PenaltyNonUser, PenaltyOptionalActivity and PenaltyOptionalTask, respectively). Moreover, high complexity and priority values further increase the rank value. The set of queries and their according rank are stored, sorted and finally presented to the administrator for revision. The query selection process only determines the order of the queries and leaves it up to the administrator to select the actual queries for disablement.

**Algorithm 1** Algorithmic details of the query selection process.

### Design of FOCALE-based control loops in the COSARA architecture

Following the FOCALE cognition model, we define three dynamic control loops with the aim of optimizing the performance of query execution. The goal of all three control loops is to keep the response time of a query group below a threshold, which corresponds with an acceptable delay. Note that the quantization of an acceptable delay is a subjective matter as it relates to how the users of the system perceive it quality (i.e., the so called Quality of Experience). Furthermore, what is acceptable also depends both on the type of application and type of operation performed (i.e., the type of query group). For our COSARA system, an acceptable delay for the most occurring query group (i.e., the microbiology query group) is up to approximately 25 seconds. This value was determined through discussions with the users of the COSARA system and based on their day-to-day experience with the system.

**Figure 7:**
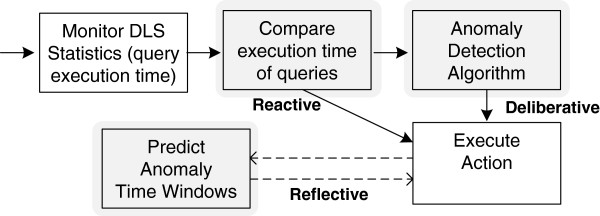
**Activities in the control flows for performance optimization.** This figure depicts the activities of each control loop. The reactive control loop reacts immediately based on the monitored query execution times. The deliberative control loop includes an anomaly detection algorithm. The reflective control loop detects temporal patterns in the query execution times. The figure shows the three control loop types.

Performance is monitored via a statistics component in the data lookup service (DLS) which stores the execution time for each query. By analyzing the execution time and deciding when a serious performance delay occurs, other queries can be disabled temporarily to ensure faster retrieval of patient’s data in the displayed client module. The method to decide and take action differs in the three presented control loops. Figure 7 depicts the activities of each control loop as explained below. Reactive control loops take immediate actions based on immediately perceived external stimuli, while the deliberative and reflective loops feature an increased level of learning: the first based on anomaly detection, while the later focuses on the clustering and detection of temporal patterns. We describe the details of all three loops in the remainder of this section.

#### Reactive control loop

The goal of the reactive control loop is to detect the occurrence of a large disruption of the system. Only if the performance of the system is severely affected, an immediate action is taken corresponding with the disablement of less important queries. As such, the reactive control loop continuously monitors the delay of page loads, as observed by the physician, in the COSARA application. To do this, the execution times of all all queries in the DLS component are monitored (FOCALE’s monitor step) and summed to a total delay as several queries will be responsible for a single page load (observe step). When this total delay is unacceptably high, denoted by the threshold *t*_*reactive*_, an alarm is raised in the control loop (Compare step). The effect of this alarm is the following: on one hand the administrator gets notified of the data problem to allow him to have a closer look of the root cause of the anomaly. On the other hand, an automatic action is also taken to ensure a graceful degradation of the system. Therefore, the automatic execution of a subset of queries, corresponding to less important data retrievals (e.g., cron jobs, side information) is disabled for a time window *W*_*reactive*_. As the total number of queries will decrease, the goal of the reactive control loop is to considerably reduce the overall perceived delay. For example, to improve the execution of microbiology samples, redundant queries of urine sediment are disabled because these queries are not shown in the module. If the physician wants to consult this urine value, a warning informs him that the query is disabled temporarily. The physician can retrieve the value by clicking a request button, in which case the value is retrieved using a duplicate urine sediment query (which can only be executed on request and is not filtered). As these urine sediment queries will only be executed when the data is actually required by the physician, the number of queries will be considerably reduced.

#### Deliberative control loop

In the deliberative control loop, decisions and actions are made using an anomaly detection algorithm. In this loop there is an explicit evaluation of the decision before acting. This control loop continuously monitors the query execution time of each individual query and groups them according to the query type. Note that, in contrast to the reactive control loop, the monitoring occurs based on each individual query and not on the grouped perceived page load. By monitoring each query type, a specific model is built that represents the typical expected query execution time of each query type. Based on this model, an anomaly detection algorithm can detect out of profile behaviour, i.e., outliers. If the share of recently detected outliers becomes abnormally high, a similar query disablement action as carried out in the reactive control loop is executed. Queries are proactively disabled when a disruption of the system is likely to occur (i.e., signalled by an increased share of abnormal individual query executions). This is in contrast to the reactive control loop where queries are disabled after a system disruption is detected. Therefore, the deliberative control loop detects patterns that typically occur just before a shortage of resources (leading to high response times) and enforces a pro-active disablement of queries to avoid the resource shortage. Additionally the deliberative control loop incorporated knowledge from a domain expert to detect the outliers. As such, the control loop consists of a training phase, where the system is trained to build the model and detect outliers, and a deployment phase, where the outliers are detected on-line and appropriate actions are taken. We discuss the algorithmic details of both phases in the remainder of this section.

##### Training phase

**Figure 8:**
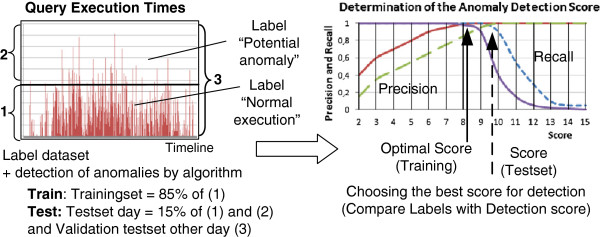
**Schematic illustration of the anomaly detection algorithm and determination of the detection score.** This schematic illustration shows the training phase and anomaly detection algorithm of the deliberative control loop. The dataset is split up in a trainingset and a test set. The query execution times are labeled with behavior normal or as an anomaly. The trainingset is used to find the optimal detection score. Precision and recall are used to choose the score. The figure illustrates the detection step in the deliberative control loop.

During the training phase, a model is built for each query type based on knowledge from a domain expert. This is shown in Figure 8. In a first step, a historical data set *D* containing the query execution times and query types over the course of a day is labeled by the domain expert (e.g., the system operator). The goal of the labelling is to select a subset *D*_*normal*_ corresponding with normal query execution times for each query type. Consequently, the subset *D*_*outlier*_ ≡ *D*∖*D*_*normal*_ corresponds with abnormal or out of profile query executions. In a second step, a random subset *D*_*train*_ ⊂ *D*_*normal*_ is taken as training set. In our case, we take 85% of random samples out of *D*_*normal*_. Based on *D*_*train*_, a model can be built of normal query execution times by calculating the mean and standard deviation of the population in *D*_*train*_. These values are then used to determine whether a random sample *x* of query execution times belongs to the calculated model or not. To do this, a z-score is calculated, which is a well known anomaly detection algorithm, as follows:

(1) $$z(x)=\frac{\left|x-\mu (D_{\text{train}})\right|}{\sigma (D_{\text{train}})}$$

Here, *μ*(*D*_*train*_) denotes the mean of *D*_*train*_, while *σ*(*D*_*train*_) is the standard deviation of *D*_*train*_. The larger the calculated z-score is, the more likely the sample *x* is to be an outlier. However, it is difficult to define a threshold for this as this depends on the distribution of the dataset, which is unknown. To address this, in a third and final step, the remaining dataset *D*_*test*_ ≡ *D*∖*D*_*train*_ is used for determining a threshold *z*_*t*_. Based on this threshold, a random sample *x* can be classified as an outlier (if *z*(*x*) > *z*_*t*_) or not. Note that *D*_*outlier*_ ⊂ *D*_*test*_: hence, *D*_*test*_ will contain both normal and out of profile query execution times. For each *x* ∈ *D*_*test*_, the z-score as defined in Equation (1) is calculated. Furthermore, for several possible values of *z*_*t*_, the samples *x* are classified and the classification is compared with the labelling of *D*_*outlier*_ and *D*_*normal*_ by the domain expert. By comparing the classification for a given *z*_*t*_ parameter configuration and the classification by the domain expert, the best *z*_*t*_ parameter configuration can be chosen. More specifically, we select the *z*_*t*_ parameter that maximises the precision and recall values, two metrics that are used to assess the accuracy of a classification system. Precision is calculated as the number of true positives (i.e. the number of true outliers) divided by the total number of elements belonging to the positive/outliers class (i.e. the number of detected outliers by the algorithm, and also including those that were listed as outlier but are not observed outlier). Recall is defined as the number of true positives divided by the total number of elements observed as outlier (i.e. the number of outliers that were detected, and also including those that were missed by the outlier detection). The result of the training phase is (i) a model for each query type, defined through the mean and standard deviation of a population of that type, that defines the normal behaviour of query execution times for that query type and (ii) a threshold *z*_*t*_ for each query type that can be used to perform an on-line outlier detection in the deployment phase.

##### Deployment phase

Once trained, the calculated model and threshold can be used to detect the occurrence of outliers on-line for each query type and act accordingly. Therefore, the deliberative control loop will continuously monitor the query execution times for each individual query and will classify each execution time as being normal or out profile according to the trained configuration. Next, the share of outliers compared to the total set of queries in the last time window *W*_*delib*_ is continuously calculated. If the calculated share exceeds a predefined threshold *s*_*outlier*_, the deliberative control loop assumes that there is a high risk of system degradation. As a reaction, it decides to execute actions that can reduce the typical query execution times (e.g., disabling other queries as discussed in Section 'Identification of candidate queries for disablement’.

#### Reflective control loop

In the reflective control loop, the long term memory is taken into account to take proactive actions (i.e, before the execution of the actual queries). The reflective loop detects temporal patterns in the occurrence of outliers and proactively disables low priority queries. In practice, the COSARA system often experiences quality degradations during peak periods (e.g., at the beginning and end of the work day of physicians). The goal of the reflective control loop is to autonomically detect these peak periods and disable the queries accordingly. Therefore, the reflective control loop has a similar goal as the deliberative control loop: it proactively disables queries to avoid high response times. The main difference is that the reflective control loop focuses on diurnal effects (i.e., patterns that can be observed on a daily basis) and disables queries for a longer time period (i.e., at least 30 minutes) based on detected historical patterns on a very long time frame (i.e., several weeks).

The reflective control loop works as follows. Based on the data set *D*_*outlier*_, constructed in the deliberative control loop using the z-score-based anomaly detection algorithm, a new data set *D*_*refl*_ is derived. As discussed, *D*_*outlier*_ contains all queries (with their response time and time of execution information), which are identified by the deliberative control loop of having abnormally high execution times (i.e., being outliers).

Based on this set of outliers, the data set *D*_*refl*_ denotes the frequency of outliers given the current time of day. To determine the frequency, we use bins of corresponding to a 30 minute time window. Hence, *D*_*refl*_ contains 48 elements. Note that, two outliers occurring on complete different days but on the same time of day will be assigned to the same bin. The relevance of this newly constructed data set is the following: we observed that peak periods often occur at the moment in time across days. This is because the physicians and nurses often use the COSARA system, as part of their routine, at the same time each day. The typical busy hours every day correspond with the start and end of every working day as well as the lunch break (around noon).

**Figure 9:**
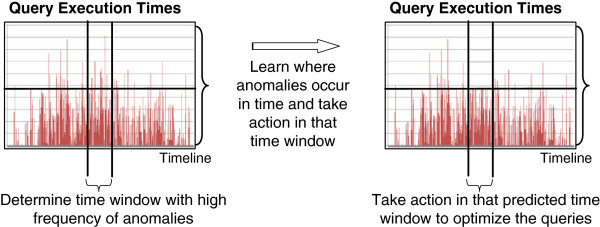
**Schematic illustration of the choice of a time window with high frequency of anomalies.** This schematic illustration shows the reflective control loop step. In a time window with a constant high frequency of anomalies an action is taken. The action prevents the high execution times in a time window. It is learned where anomalies occur in time and the action is taken in that specific time window.

By deriving the data set *D*_*refl*_ we can identify the aforementioned busy hours, as they will correspond with a high number of outliers in *D*_*outlier*_ and thus in large values for the frequency in that time zone. In order to detect the highest values, we apply the same anomaly detection algorithm as discussed above: we calculate the z-score as well as the optimal z-score threshold and classify a time window as an outlier if its z-score is above the defined threshold. This process is illustrated in Figure 9.

In this context, the detection of an outlier signifies a time window of 30 minutes, where the deliberative control loop has found an abnormal amount of high response times. Hence, this builds further on the knowledge learned in the previous control loop. If such a time window is identified as an outlier, the queries are disabled during that time window. This is done pro-actively on every day in the future, until the reflective control loop no longer flags this as a busy period.

## Results and discussion

In this section we study the influence of the actions of the FOCALE based reactive and deliberative control loops on the query execution time. The executed queries of a random day in January 2012 were taken and executed again in a test environment, as described in the evaluation setup. The duration of each COSARA query was measured. In these experiments we evaluated the impact of an immediate action in reactive control loop, the decision to take action by the described anomaly detection algorithm in the deliberative loop and its impact.

### Evaluation setup

**Figure 10:**
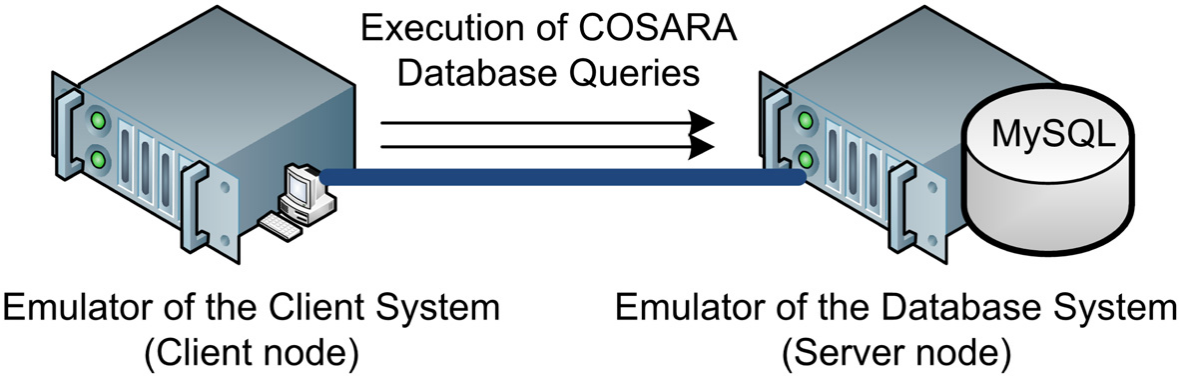
**Setup of COSARA client and server on nodes of the generic test environment.** The generic test environment consists of an emulator for the COSARA client and for the COSARA server. The client system executes the queries on the COSARA database, a MySQL database. With this experimental setup, as shown on the figure, the COSARA behavior is replicated and the effect of the applied control loops can be measured.

The COSARA platform is set up in the real life production environment of the Ghent University Hospital. By logging the performed queries in the production environment, we were able to emulate the query executions on the COSARA database in a test environment and evaluate the impact of optimization without interfering in the clinical workflow of the production environment. Due to the sensitivity of the patients’ electronic health records and the medical decisions based on this data, we have replicated the COSARA database queries on test servers. In this section, we detail the used experimental setup to replicate COSARA’s behavior. In the experiments we measure the query execution times by replicating the COSARA queries in a multi-threaded application. By replaying the queries again with a specific action, we are able to evaluate the impact of action by the control loops on the query execution time. In the action we disable the queries COSARA UrineSediment and Identification as these were identified as less important by our query selection process. For executing the query selection process, we set the penalty parameters PenaltyUser, PenaltyOptionalActivity and PenaltyOptionalTask to 10, 5 and 3, respectively. The UrineSediment queries retrieve the urine sediment value. The Identification queries retrieve changes made to the identification. Both queries are not important when the physician is actually displaying the patients’ microbiology. The results include the average query execution time of the query executions. These percentiles show the worst execution times. These executions cause delay on the physicians screen while displaying microbiology samples. The experiments were carried out on one core Intel Xeon E5620 processor with 2.40 GHz and 3.0 GB RAM. We emulated the client and server interaction by replicating the COSARA queries of the trace log. As such, it shows behavior identical to the query executions in production environment. Figure 10 shows the test setup. On the server node a MySQL database server 5.0.51 is running and on the client node we rerun the real executed queries and measure its execution time and the improvements by the actions in the presented control loops.

### Performance evaluation of the control loops

#### Performance evaluation of the reactive control loop

In the reactive control loop an action is immediately taken if the performance is severely affected. We set parameter *t*_*reactive*_ to 90,000 ms and parameter *W*_*reactive*_ to 2 minutes. The value *t*_*reactive*_ corresponds to the execution time, which is considered very obstructive by the COSARA user. It is expected that similar high execution times are prevented by setting a time window of 2 minutes for the action.

**Figure 11:**
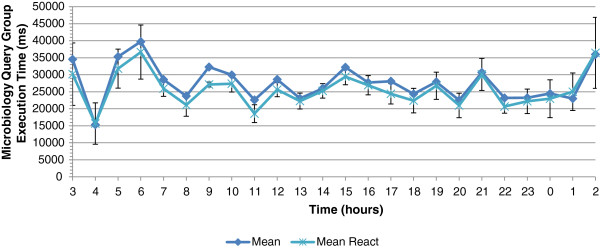
**Average execution time of microbiology query group without management and with actions by reactive loop.** This figure shows the average execution time of the microbiology query group over 24 hours without autonomic management and with actions taken by the reactive control loop. The average exeution time is 27 s without autonomic action and 24 s with the reactive action.

Figure 11 depicts the average execution time of a COSARA microbiology group without autonomic management actions and with the action taken in the reactive control loop. The average execution time is 27,308 ms without autonomic action and 24,956 ms with the reactive action. The reactive control loop provides a gain of 8.61% or 2,352 ms on the average query execution time of the microbiology query group. The impact of the control loops on the query execution times of microbiology queries group is analysed per hour, as shown in Figure 11. The highest gain is observed in the period from 9 to 10 am with a reduction of 15.92% on the average execution time.

#### Performance evaluation of the deliberative control loop

**Figure 12:**
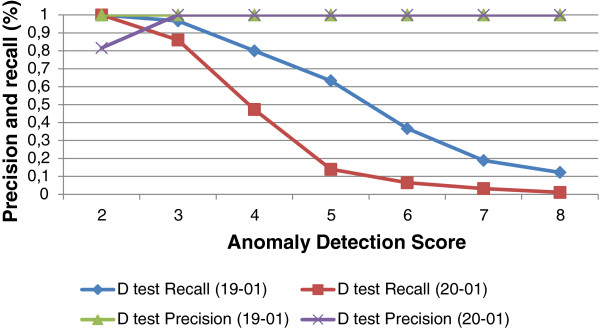
**Example of the determination of anomaly detection score based on highest precision and recall.** The figure shows an example of the determination of anomaly detection score z for query COSARA Microbiology Samples based on highest precision and recall.

**Figure 13:**
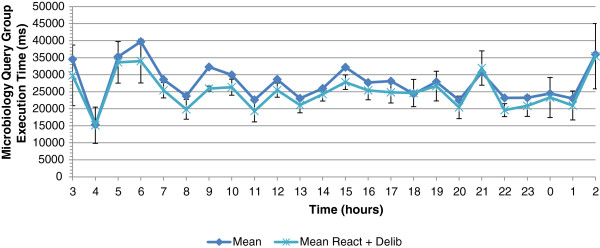
**Average execution time of microbiology without management and with actions taken by reactive, deliberative loops.** The figure shows the average execution time of the microbiology query group over 24 hours without autonomic management and with actions taken by the reactive and deliberative control loops.

The reactive control loop already offers an important gain but optimizations are still possible. In the deliberative control loop the decision and action are made using an anomaly detection algorithm. In this section we evaluate the effect of the deliberative control loop on the query execution times. First we determine the detection score *z*_*t*_, as discussed in Section 'Deliberative control loop’. We varied the score from 2 to 8. Figure 12 shows an example of the determination of the anomaly detection score with precision and recall (based on training set, data log of January, 19, 2012). Based on this we set the anomaly detection score *z*_*t*_ to 2 because of its highest precision and recall. The figure also shows the precision and recall of *D*_*test*_ (log results of January, 20, 2012, the test set). We analysed the queries which retrieve microbiology samples, cultures, antibiogram and analyses and determined the scores. Then, we set *W*_*delib*_ to a time period of 1000 ms in which queries were executed. The parameter *s*_*outlier*_ was set to 20% to obtain the best share of outliers. The action, identical to the disabling of queries in the reactive loop, was performed when *s*_*outlier*_ was exceeded. Figure 13 shows the average query execution time without autonomic management and with actions taken in the reactive and deliberative control loop. We compared each observation with its original execution time. The combination of the reactive and deliberative control loop performs better than only the reactive one. The combination gives a reduction of 2,980 ms or 10.92% to the original average execution time without management. The combined actions give an average execution time of 24,327 ms. The highest reduction of the average execution time is observed in the period from 9 to 10 am with a reduction of 19.69%.

#### Performance evaluation of the reflective control loop

**Figure 14:**
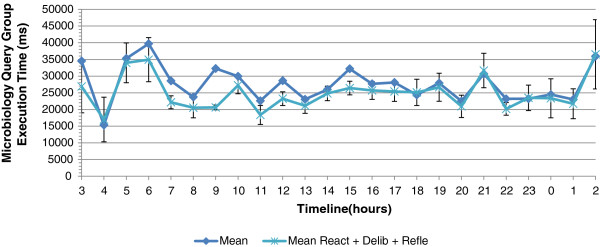
**Average execution time of microbiology without management and with actions taken by reactive, deliberative, reflective loops.** The figure shows the average execution time of the microbiology query group over 24 hours without autonomic management and with actions taken by the reactive, deliberative and reflective control loops.

In the reflective control loop, queries are disabled proactively during peak periods of high query execution times. In the detected time intervals (7h to 7h30), (9h to 10h), (12h to 12h30) and (16h to 17h), we disable the UrineSediment and Identification query proactively. The combination of the reactive, deliberative and reflective actions affects the query group execution times additionally during the selected time intervals. Figure 14 shows the average query group execution time per hour. The microbiology group query execution time without autonomic management is 27,308 ms, where the execution time with the combination of reactive, deliberative and reflective actions is 23,747 ms or a reduction with 13.04%. In absolute values, the above results show that we can keep the average response times below 25 seconds with the exception of peaks caused by nightly background tasks, which are not perceivable by the users. As discussed above and based on feedback from the users of the COSARA system, this is an acceptable response time. In the selected time interval from 9 to 10 am, a reduction of 36.11% is observed on the average exeution time by applying the three control loops where the reactive gave only a reduction of 15.92% and the reactive and deliberative gave a reduction of 19.69%. The reflective control loop acts proactively during predicted peak periods and hence prevents high query execution times.

### Discussion

The proposed solution introduces an alternative to simply increasing the amount of resources by upgrading the physical infrastructure. The control loop solution is viable as the response times in query processing experience important peak periods during certain moments in time (e.g., the beginning and end of the day). Upgrading the physical infrastructure to accommodate these peaks is possible, but at the same time costly as the infrastructure would often be idle during less busy periods. As our solution takes care of the abnormally high peaks in response time, the result is a more flat behaviour of response time over the day. Therefore, if the system’s usage would increase further, deploying alternative solutions such as database replication in combination with our proposed solution will be more advantageous.

**Figure 15:**
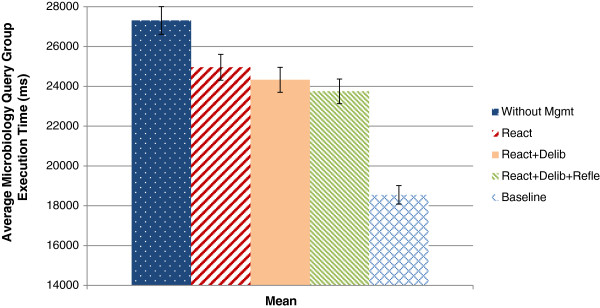
**Comparison of the average microbiology query execution time without and with autonomic management.** The average microbiology query execution time is compared. The figure shows the query exeuction time of the reactive, deliberative and reflective control loops. It shows the affected average query execution times with the combined control loops and the baseline over 24 hours.

**Figure 16:**
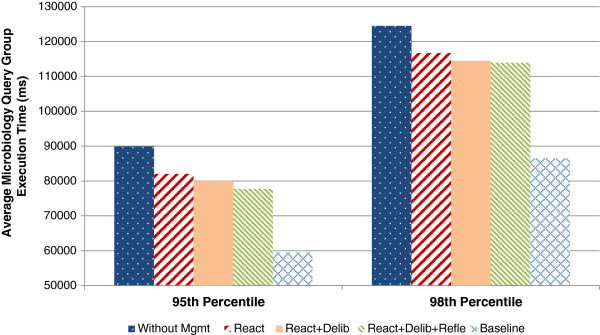
**Comparison of the 95th and 98th percentile of microbiology query execution without and with management.** Comparison of the 95th and 98th percentile of the microbiology query execution time without and with autonomic management of the reactive, deliberative and reflective control loops. The figure shows the affected 95th and 98th percentile query execution times with the combined control loops and the baseline over 24 hours.

Figure 15 and Figure 16 summarize all the evaluation results. Figure 15 compares the average query group execution times. The reactive control loop reduces the average execution time by 8.61%. The combination of the actions of the reactive and deliberative control loop reduce the average execution time by 10.92%. The combination with the reflective control loop affects the average execution time with 13.04%. By disabling the queries during the whole day the baseline is set. Although this shows the highest possible gain, the queries COSARA UrineSediment and Identification are disabled completely. Compared to the baseline, the control loops provide a reduction of more than one third of the possible improvement. The baseline showed a difference of 32.09% compared to the average execution time without actions. We also compared the effect on the 95th and 98th percentile, as shown in Figure 16. The reactive control loop, reactive and deliberative control loop, and reactive, deliberative and reflective show reductions of 8.79%, 11.04% and 13.57% respectively in the 95th percentile. In the 98th percentile the execution time is reduced by 6.27%, 8.07% and 8.50% respectively.

Overall, the solution has a high scalability for several reasons. All three control loops rely on detecting peaks based on summarized data. This means that the memory consumption does not grow linearly with an increasing number of users of the system. Furthermore, the control loops introduce only a marginal overhead in terms of computational complexity. Finally, in the design of all three loops, care has been taken to maintain a good scalability. For example, the reactive control loop was deliberately kept relatively simple in terms of computational complexity and memory consumption as it needs to run at a very high frequency (i.e., in the order of seconds). Control loops which run on a more daily basis (e.g., the reflective control loop) are allowed to introduce a higher complexity. Note that, as the reflective control loop mainly relies on clustering, it also has a high scalability as the number of users increases.

## Conclusions

This paper presents the extension of the existing health care platform COSARA in the ICU with autonomic control loops. The introduced control loops provide an automated mechanism to detect low performance and to take action, thereby limiting human technical interventions. The monitoring of the execution times of the data queries of this real life intensive care platform allow the investigation of low performance. A reactive, deliberative and reflective control loop have been proposed to optimize the data query performance and thus the page load of the microbiology module. In the reactive control loop the action is immediately taken when the performance of the system is affected. The action disables less important queries not relevant for the display of microbiology data. In the deliberative control loop we use an anomaly detection algorithm with an explicit evaluation of the decision before the action is taken. In the reflective control loop, proactive actions are taken after temporal patterns of outliers are detected. We evaluated the impact of the reactive, deliberative and reflective control loop on the query execution of the microbiology data. The results show a time reduction of 8.61% by the reactive control loop on the average query execution times. The addition of the deliberative control loop reduced the average query execution time by 10.9% and by combining the three control loops the average execution time was reduced by 13.04%.

## Endnotes

^a^ Resource Description Framework (RDF) - http://www.w3.org/RDF/

^b^ OWL Web Ontology Language Overview - http://www.w3.org/TR/owl-features/

^c^ IntelLEO Workflow ontology - http://www.intelleo.eu/ontologies/workflow/spec/

## Competing interests

The authors declare that they have no competing interests.

## Authors’ contributions

The work presented was carried out in collaboration between all authors. KS and SL carried out the study of the control loops on the COSARA platform, participated in the design and development of the control loops as described in this paper and drafted the manuscript. FDT and JD supervised the study, participated in the design and coordination and helped to draft the final manuscript. All authors read and approved the final manuscript.

## Pre-publication history

The pre-publication history for this paper can be accessed here:

http://www.biomedcentral.com/1472-6947/13/120/prepub
